# Transmission of Atypical Bovine Prions to Mice Transgenic for Human Prion Protein

**DOI:** 10.3201/eid1412.080941

**Published:** 2008-12

**Authors:** Vincent Béringue, Laëtitia Herzog, Fabienne Reine, Annick Le Dur, Cristina Casalone, Jean-Luc Vilotte, Hubert Laude

**Affiliations:** Institut National de la Recherche Agronomique, Jouy-en-Josas, France (V. Béringue, L. Herzog, F. Reine, A. Le Dur, J.-L. Vilotte, H. Laude); Istituto Zooprofilattico Sperimentale del Piemonte, Liguria e Valle d’Aosta, Turin, Italy (C. Casalone)

**Keywords:** prions, BSE, PrP, strains, transgenic mice, dispatch

## Abstract

To assess risk for cattle-to-human transmission of prions that cause uncommon forms of bovine spongiform encephalopathy (BSE), we inoculated mice expressing human PrP Met^129^ with field isolates. Unlike classical BSE agent, L-type prions appeared to propagate in these mice with no obvious transmission barrier. H-type prions failed to infect the mice.

The epizootic of bovine spongiform encephalopathy (BSE) is under control in European countries >20 years after the first cases were diagnosed in the United Kingdom. Thus far, BSE is the only animal prion disease known to have been transmitted to humans, leading to a variant form of Creutzfeldt-Jakob disease (vCJD) (*1*). The large-scale testing of livestock nervous tissues for the presence of protease-resistant prion protein (PrP^res^) has enabled assessment of BSE prevalence and exclusion of BSE-infected animals from human food (*2*). This active surveillance has led to the recognition of 2 variant PrP^res^ molecular signatures, termed H-type and L-type BSE. They differ from that of classical BSE by having protease-resistant fragments of a higher (H) or a slightly lower (L) molecular mass, respectively, and different patterns of glycosylation (*3*–*5*). Both types have been detected worldwide as rare cases in older animals, at a low prevalence consistent with the possibility of sporadic forms of prion diseases in cattle (*6*). Their experimental transmission to mice transgenic for bovine PrP demonstrated the infectious nature of such cases and the existence of distinct prion strains in cattle (*5*,*7*–*9*).

Like the classical BSE agent, H- and L-type prions can propagate in heterologous species (*7*–*11*). Thus, both agents are transmissible to transgenic mice expressing ovine PrP (VRQ allele). Although H-type molecular properties are conserved on these mice (*9*), L-type prions acquire molecular and neuropathologic phenotypic traits undistinguishable from BSE or BSE-related agents that have followed the same transmission history (*7*). Similar findings have been reported in wild-type mice (*8*). An understanding of the transmission properties of these newly recognized prions when confronted with the human PrP sequence is needed. In a previous study, we measured kinetics of PrP^res^ deposition in the brain to show that L-type prions replicate faster than BSE prions in experimentally inoculated mice that express human PrP (*7*). In a similar mouse model, the L-type agent (alternatively named BASE) was also shown to produce overt disease with an attack rate of ≈30% (*12*). However, no strict comparison with BSE agent has been attempted. As regards the H-type agent, its potential virulence for mice that express human PrP Met^129^ remains to be assessed. We now report comparative transmission data for these atypical and classical BSE prions.

## The Study

**Figure 1:**
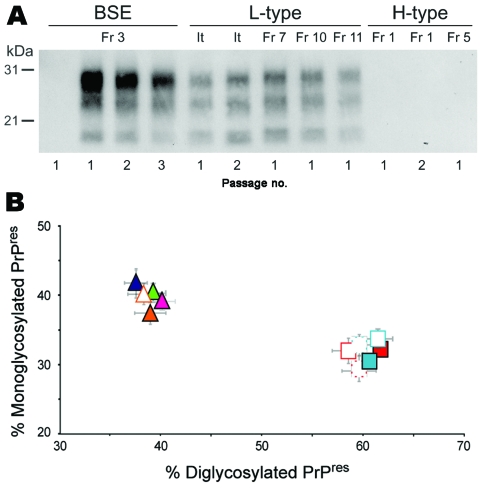
Protease-resistant prion protein (PrP^res^) in the brains of human PrP transgenic mice infected with atypical or zoonotic bovine spongiform encephalopathy (BSE) agents. A) Representative Western blot analysis of PrP^res^ extracted (for detailed protocol, see *7*) from brain homogenates of mice at terminal stage of disease or at end of lifespan after serial transmission of atypical (L-type and H-type) or classical BSE isolates. The amount of equivalent brain tissue loaded onto the gels was 0.01 mg (BSE; Fr3 isolate, 2nd and 3rd passage), 0.3 mg (L-type and 1st passage of BSE), and 10 mg (PrP^res^-negative samples). Anti-PrP monoclonal antibody Sha31 was used for PrP^res^ detection. Immunoreactivity was determined by chemiluminescence. B) Ratio of diglycosylated and monoglycosylated PrP^res^ species in the brains of mice after serial transmission of L-type or BSE isolates (data plotted as means ± standard error of the mean). Primary passage of L-type isolates are represented as triangles (orange, It; blue, Fr7; green, Fr10; pink, Fr11) and BSE as squares (light blue, Fr3; red, Ge). Passages are indicated by unfilled symbols of the same color (solid line, second passage; broken line, third passage). The ratio was determined after acquisition of PrP^res^ chemiluminescent signals with a digital imager as previously described (*7*). Note the distinct glycoform ratio between L-type and BSE groups. It, Italy; Fr, France; Ge, Germany.

The bovine isolates used in this study have been previously described; they all exhibited high infectivity levels in bovine PrP mice (*4*,*7*,*9*). The equivalent of 2 mg of infected bovine brain tissue was injected intracerebrally into *tg650* mice. This line of mice overexpresses (≈6-fold) human PrP with methionine at codon 129 (Met^129^) on a Zurich mouse PrP null background and has been shown to be fully susceptible to vCJD agent (*13*). The resulting transmission data available to date are summarized in the Table. The primary transmission of classical BSE isolates was inefficient as judged by the absence of clear neurologic signs and by Western blot detection of PrP^res^ in the brain of only 4/25 inoculated mice. The PrP^res^ banding pattern was essentially similar to that of vCJD (low molecular mass fragments and predominance of diglycoform species; Figure 1).

Secondary passages were performed by using PrP^res^-negative or PrP^res^-positive individual mouse brains. Every time brain homogenate from an aged mouse (>630 days of age) was inoculated, transmission was observed in >80% of the mice, as determined by clinical signs and PrP^res^ accumulation. The mean survival time was ≈600 days (Table; additional data not shown). By the third passage, mean survival time approached ≈500 days, as is usually observed with vCJD cases (Table; *13*). The vCJD-like PrP^res^ profile was conserved in all 50 positive brains analyzed (Figure 1). Transmission of L-type isolates to *tg650* mice produced markedly different results. First, the 4 L-type isolates induced neurologic disease in almost all mice; survival times averaged 600–700 days (Table). Second, PrP^res^ accumulated in the brain of all 33 mice analyzed. The molecular profile was distinct from BSE or vCJD; prominent monoglycosylated PrP^res^ species resembled those found in cattle (Figure 1). Third, we found neither shortening of the survival time on subpassage (1 isolate tested, 2 brains) (Table; data not shown) nor change in PrP^res^ profile (Figure 1).

**Table:** Transmission of classical and atypical BSE isolates to transgenic mice expressing human prion protein (Met^129^)*

Isolate	Origin (identification no.)	1st passage			2nd passage			3rd passage	
		Total no. affected†	Mean survival time‡		Total no. affected†	Mean survival time‡		Total no. affected†	Mean survival time‡
BSE	France (3)	1/6^§^	872		6/7	568 ± 65		8/8	523 ± 22
	France (3)	2/6	627, 842						
	Germany¶	1/4§	802		6/6	677 ± 54		8/8	555 ± 24
	Italy (128204)	0/5	606–775						
	Belgium	0/4	696–829						
L-type	Italy (1088)	9/9	607 ± 23		11/11	653 ± 13			NA
	France (7)	7/7	574 ± 35		0/8#	>450			
	France (10)	8/8	703 ± 19						
	France (11)	9/9	647 ± 26						
H-type	France (1)	0/6	376–721		0/7	350–850			NA
	France (2)	0/6	313–626		0/8	302–755			NA
	France (5)	0/10	355–838			NA			

*BSE, bovine spongiform encephalopathy; NA, not available (experiments still ongoing). †Mice with neurologic signs and positive for protease-resistant prion protein by Western blotting. ‡Days ± SE of the mean. For mice with negative results, only the range of survival time is given. §First passage performed on hemizygous mice. Note that the primary transmission of France (3) isolate was performed on both hemizygous and homozygous mice. ¶One passage on transgenic mice expressing bovine prion protein (*7*). #Ongoing experiment.

In sharp contrast, BSE H-type isolates failed to transmit disease to or even infect *tg650* mice. None of the inoculated mice had a detectable level of PrP^res^ in the brain (22 analyzed). Secondary passages were performed with brains of mice that died at various time points. All inoculated mice survived, and none showed a PrP^res^ signal in the brain (15 mice analyzed).

**Figure 2:**
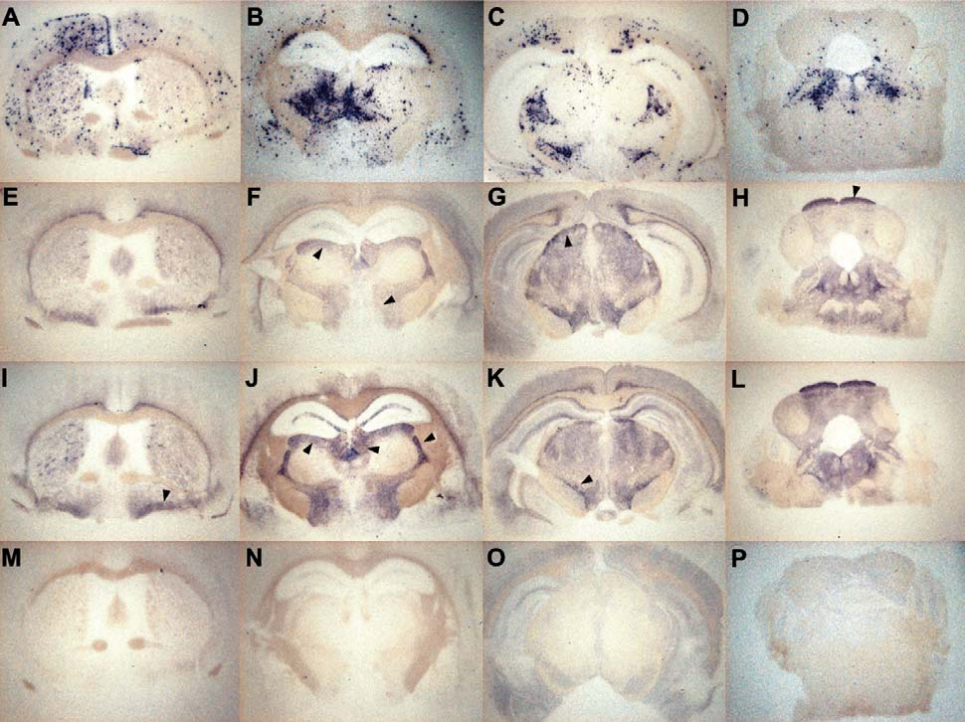
Representative histoblots in 4 different anteroposterior sections showing the distribution of disease-specific PrP^res^ deposits in the brains of *tg650* mice infected with bovine spongiform encephalopathy (BSE) or L-type BSE. Panels A–D show infection with BSE (second passage of France 3). Panels E–H show infection with L-type BSE (first passage of France 7). Panels I–L show infection with L-type BSE (second passage of Italy). Panels M–P show brain sections of an age-matched, mock-infected mouse, euthanized while healthy at 700 days postinfection, for comparison. Note the differing aspect and distribution of PrP^res^ deposits between brain of mice infected with BSE and BSE-L (arrowheads). Assignment of the positive brain regions has been made according to a mouse brain atlas after digital acquisition.

To further compare the behavior of the 3 bovine prions in *tg650* mice, we examined the regional distribution and intensity of PrP^res^ deposition in the brain (*14*). Histoblot analyses (3 brains per infection) were performed on primary (L-type, H-type) and secondary passages (L-type, H-type, BSE). As shown in Figure 2, L-type and BSE agents showed clear differences according to both the aspect and localization of the PrP deposits. Granular PrP deposits were scattered throughout the brain with BSE, as has been previously observed with vCJD (*13*). The ventral nuclei of the thalamus, cerebral cortex, oriens layer of the hippocampus, and raphe and tegmentum nuclei of the brain stem were strongly stained. With BSE-L, the staining was finer and essentially confined to the habenular, geniculate, and dorsal nuclei of the thalamus; the lateral hypothalamus; the lacunosom moleculare layer of the hippocampus; the superficial gray layer of the superior colliculus; and the raphe nuclei of the brain stem. Finally, PrP^res^ could not be detected on brain sections from mice inoculated with H-type isolates (data not shown), thus confirming the Western blot data.

## Conclusions

We found that atypical L-type bovine prions can propagate in human PrP transgenic mice with no significant transmission barrier. Lack of a barrier is supported by the 100% attack rate, the absence of reduction of incubation time on secondary passage, and the conservation of PrP^res^ electrophoretic profile. In comparison, transmission of classical BSE agent to the same mice showed a substantial barrier. Indeed, 3 passages were necessary to reach a degree of virulence comparable to that of vCJD agent in these mice (*13*), which likely reflects progressive adaptation of the agent to its new host. At variance with the successful transmission of classical BSE and L-type agents, H-type agent failed to infect *tg650* mice. These mice overexpress human PrP and were inoculated intracranially with a low dilution inoculum (10% homogenate). Therefore, this result supports the view that the transmission barrier of BSE-H from cattle to humans might be quite robust. It also illustrates the primacy of the strain over PrP sequence matching for cross-species transmission of prions (*15*).

Extrapolation of our data raises the theoretical possibility that the zoonotic risk associated with BSE-L prions might be higher than that associated with classical BSE, at least for humans carrying the Met^129^ PrP allele. This information underlines the need for more intensive investigations, in particular regarding the tissue tropism of this agent. Its ability to colonize lymphoid tissues is a potential, key factor for a successful transmission by peripheral route. This issue is currently being explored in the *tg650* mice. Although recent data in humanized mice suggested that BSE-L agent is likely to be lymphotropic (*12*), preliminary observations in our model suggested that its ability to colonize such tissues is comparatively much lower than that of classical BSE agent.
